# Activity of antimicrobial photodynamic therapy against a cariogenic biofilm composed of a microbial consortium

**DOI:** 10.1117/1.JBO.30.11.118001

**Published:** 2025-11-06

**Authors:** Bruna Alves Thurler, Thamyris Py Domingos Faial Santos, Paula Carvalho Motta, Gabriella Lorena Dias Pereira, Gabriela Ceccon Chianca, Helvecio Cardoso Correa Povoa, Karla Bianca Fernandes da Costa Fontes, Natalia Lopes Pontes Povoa Iorio

**Affiliations:** a Universidade Federal Fluminense (UFF), Nova Friburgo Institute of Health, Experimental and Applied Microbiology Laboratory, Nova Friburgo, Brazil; b Universidade Federal Fluminense (UFF), School of Medicine, Graduate Program in Pathology, Niterói, Brazil; c Faculdade Pitágoras, Teixeira de Freitas, Brazil; d Universidade Federal Fluminense (UFF), Nova Friburgo Institute of Health, Graduate Program in Dentistry, Nova Friburgo, Brazil; e Universidade Federal Fluminense (UFF), Biomedical Institute, Graduate Program in Applied Microbiology and Parasitology, Niterói, Brazil; f Universidade Federal Fluminense (UFF), Nova Friburgo Institute of Health, Department of Specific Formation, Nova Friburgo, Brazil

**Keywords:** antimicrobial photodynamic therapy, biofilms, microbiology, photochemotherapy

## Abstract

**Significance:**

Dental caries is a polymicrobial condition derived from microbial biofilm. There is a lack of studies addressing antimicrobial photodynamic therapy (aPDT) activity against a cariogenic multispecies biofilm.

**Aim:**

We aim to evaluate the activity of aPDT against a cariogenic biofilm composed of a microbial consortium.

**Approach:**

Equal parts of *Streptococcus mutans*, *Lactobacillus rhamnosus*, and *Candida albicans* were used to form a microbial inoculum containing $∼10^{7}$ colony-forming units/mL, which was placed on cellulose acetate membranes to form biofilm. After biofilm formation, the seven groups, each containing four membranes, were treated as follows: laser 1 J (G1); laser 4 J (G2); photosensitizer methylene blue (G3); photosensitizer + laser 1 J (G4); photosensitizer + laser 4 J (G5); chlorhexidine as positive control (G6); and distilled water (G7).

**Results:**

The number of viable microbial cells per biofilm varied between $1.40\times 10^{8}$ (G5) and $7.28\times 10^{8}$ (G1), whereas the negative control group (G7) reached $1.38\times 10^{9}$. Compared with G7, all groups presented a reduction, with the percentage varying from 47.05% (G1) to 89.85% (G5). However, G5 (photosensitizer + laser 4 J) was the only group to present a statistical reduction (p<0.05).

**Conclusion:**

aPDT represents an important antibiofilm adjunct therapy, resulting in a significant reduction in microbial cells within a cariogenic biofilm model.

## 1 Introduction

Antimicrobial photodynamic therapy (aPDT) results in photochemical reactions that lead to microbial cell death.^1^ The production of reactive oxygen species through the combination of a photosensitizer, light activation, and molecular oxygen causes the destruction of target cells due to type I and/or type II reactions. Type I pathway occurs through the transference of electrons or hydrogen atoms once the photosensitizer absorbs photons, initiating free-radical chain reactions and producing peroxides, superoxide anion, and hydroxyl radicals. Type II pathway involves energy transfer to ground state oxygen (O21), followed by return of the photosensitizer to its ground state; this pathway generates singlet oxygen, which efficiently interacts with many biomolecules.^2^ This therapy presents no significant cytotoxic effects to human cells,^3^ and its effects have been evaluated in many studies, including dental ones,^4^ such as in root canal treatment,^5^ peri-implantitis,^6^ orthodontics,^7^ and carious lesions.^8^^,^^9^

Dental caries is a polymicrobial condition characterized by the demineralization of the tooth surface due to organic acids from bacterial carbohydrate metabolism.^10^^,^^11^ Many bacterial species that may play an important role in dental caries have been described, such as *Streptococcus mutans* and *Lactobacillus*.^12^ Furthermore, clinical evidence has shown that the coexistence of bacteria and fungi in the oral cavity may increase susceptibility to infection. An assortment of oral bacteria can interact with *Candida albicans*, affecting biofilm development and contributing to the virulence of many oral diseases, including dental caries.^13^

Even though *S. mutans* does not act alone in dental caries development, it primarily inhabits biofilms on tooth surfaces. This microorganism also has the ability to adapt to multiple environmental changes within dental biofilm and has been investigated as a possible target to prevent dental caries.^10^ *Lactobacillus* spp. play a greater role in dental caries progression than in its initial stage.^14^ The yeast *C. albicans* is able to interact with many oral microorganisms in an interdependent, mutually beneficial way.^15^ The association between *C. albicans* and *S. mutans* via production of exopolysaccharides increases both fungal and bacterial carriage; synergistically, these microorganisms can increase the virulence of biofilms.^16^

Several studies have already evaluated the antibiofilm action of different light sources^17^^,^^18^ and photosensitizers,^19^^–^^21^ as well as aPDT;^20^^–^^23^ nevertheless, there is still a lack of studies addressing antimicrobial photodynamic activity against a cariogenic multispecies biofilm.

The aim of this study was to evaluate the *in vitro* activity of aPDT against a cariogenic biofilm formed by three microorganisms. The null hypothesis tested was that there would be no microbial reduction in a biofilm composed of a cariogenic consortium treated with aPDT.

## 2 Materials and Methods

### 2.1 Microbial Strains

Microbial samples of *S. mutans* American Type Culture Collection (ATCC) 25175, *Lactobacillus rhamnosus* ATCC 9595, and *C. albicans* ATCC 10231 were used in this study.

### 2.2 Sample Size Calculation

The sample size was determined based on a previous study of Gong et al.,^24^ using means and standard deviations of viable cells of *S. mutans* and *C. albicans* present in a multispecies cariogenic biofilm on hydroxyapatite discs before ($3.78\times 10^{5}\pm 1.17\times 10^{5}$ and $2.4\times 10^{4}\pm 0.5\times 10^{4}$) and after ($1.6\times 10^{5}\pm 1.9\times 10^{4}$ and $1.4\times 10^{4}\pm 0.2\times 10^{4}$) light-emitting diode (LED) irradiation (18 J). One-tailed independent sample t-test was used in the statistical program BioEstat 5.3 software (Institute for Sustainable Development Mamiraua, Tefe, AM, Brazil, a freeware available at Ref. 25) with a 5% level of significance and 95% power. Thus, the sample reached a minimum of three membranes per experimental group. The formula of this test is also available at the BioEstat 5.3 software “Help” section.

### 2.3 Inoculum

**Fig. 1:**
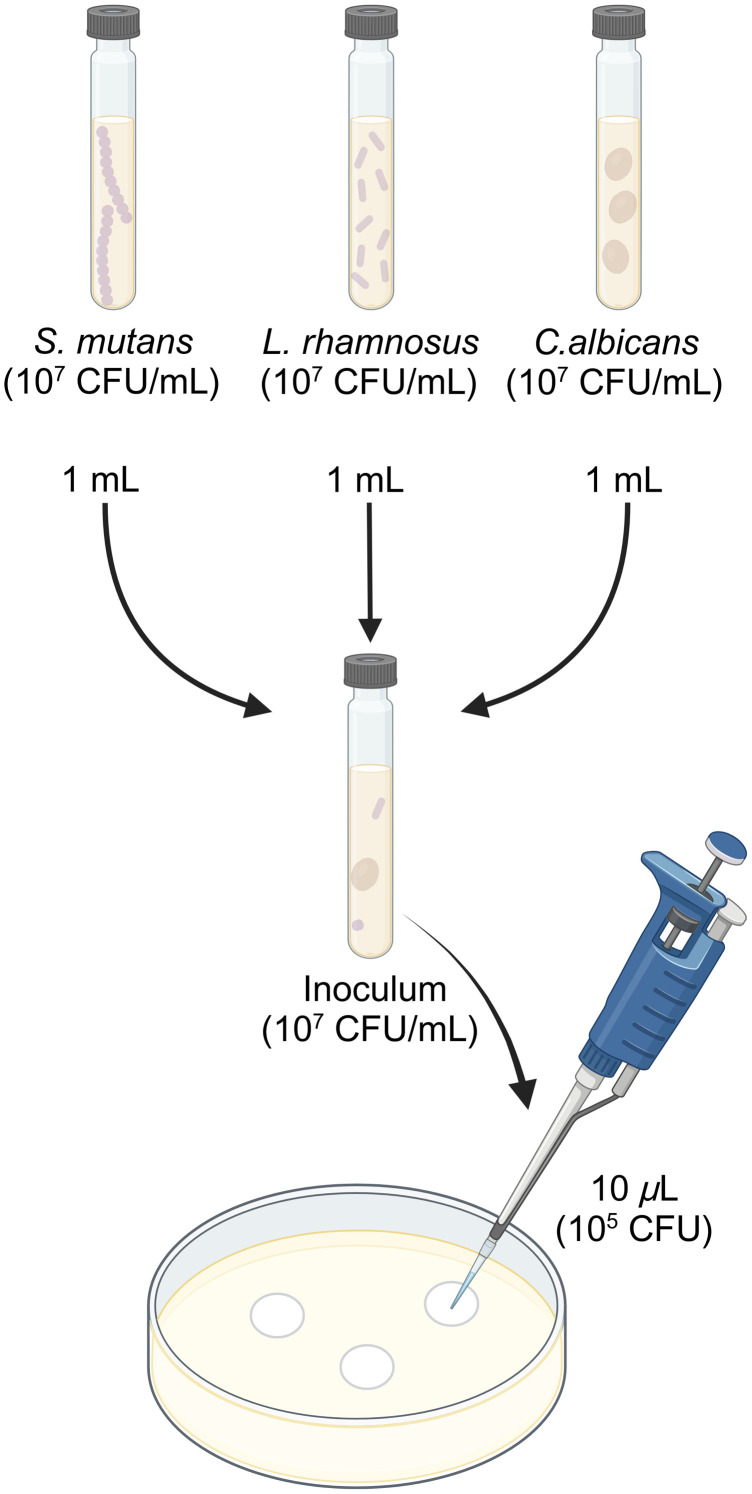
Inoculum and biofilm preparation. CFU, colony-forming units. (Created with Ref. 26).

The samples were grown on Brain Heart Infusion (BHI) agar (OXOID, Hampshire, England) at 36°C for 24 h. *S. mutans* and *L. rhamnosus* were incubated in a candle jar to create a microaerophilic condition, whereas *C. albicans* was kept in an aerobic atmosphere. After this period, microbial colonies were transferred to a 0.9% sterile saline solution until reaching an optical density (O.D.) of 0.15 (*S. mutans*), 0.20 (*L. rhamnosus*), and 1.99 (*C. albicans*), at 520 nm (Libra S2 Colorimeter, Biochrom, Cambridge, England), which corresponds to $∼10^{8}$ colony-forming units (CFU)/mL for the bacterial samples and $10^{7}$ for the fungal one. Each bacterial sample was submitted to a decimal dilution, and 1 mL of each microbial solution (containing $∼10^{7}\;\mathrm{CFU}/\mathrm{mL}$ of each microorganism) was transferred to a single sterile tube, composing the microbial inoculum used in this study (Fig. 1).

### 2.4 Biofilm

To form the biofilm consortium, sterile 0.2  μm cellulose acetate filters with a 0.13 mm diameter (Sartorius Stedim Biotech, Gottingen, Germany) were placed onto BHI agar (OXOID) supplemented with 2% sucrose (ISOFAR, Rio de Janeiro, Brazil). Each membrane received 10  μL of the microbial inoculum, carrying $10^{5}\;\mathrm{CFU}/3$ of *S. mutans*, $10^{5}\;\mathrm{CFU}/3$ of *L. rhamnosus*, and $10^{5}\;\mathrm{CFU}/3$ of *C. albicans*, totalizing $10^{5}\;\mathrm{CFU}$ of microorganisms per disk (Fig. 1). The set was then incubated in aerobic conditions at 36°C for 24 h.

### 2.5 Treatments

After biofilm formation, the membranes were treated with the photosensitizer 0.01% (100  μg/mL) methylene blue solution (compounded by Fórmula & Ação, São Paulo, Brazil) with pre-irradiation time of 5 min and/or irradiated with low-level laser therapy by using a red diode laser (Laser Duo, MMOptics, São Carlos, Brazil) with 660 nm wavelength, indium–gallium–aluminum–phosphide (InGaAlP), with 100 mW of output power, 1 J of output energy, $33.3\;J/{\mathrm{cm}}^{2}$ of energy density and exposure time of 10 s or 4 J of output energy, $133.3\;J/{\mathrm{cm}}^{2}$ of energy density and 40 s of exposure time, at a distance of 2 mm. The power of the red laser device was checked using the LaserCheck (MMOptics) to confirm the potency of 100±20  mW.

Chlorhexidine 0.12% was used as a positive control (1 min) and sterile distilled water as the negative control (1 min), totalizing seven groups, as follows: group 1 (G1): laser (1 J); group 2 (G2): laser (4 J); group 3 (G3): photosensitizer; group 4 (G4): photosensitizer + laser (1 J); group 5 (G5): photosensitizer + laser (4 J); group 6 (G6): chlorhexidine; and group 7 (G7): distilled water. Each group was composed of four membrane disks.

Immediately after treatment, the disks were washed five times with sterile saline to remove planktonic cells. Each disk was transferred to a tube containing 1 mL of sterile saline and then subjected to three 8-min cycles of 42 Hz ultrasonic waves (Bio Art, São Paulo, Brazil) aiming biofilm detachment. Serial dilutions ($10^{-1}$ to $10^{-7}$) were performed, and 50  μL of dilutions from $10^{-3}$ to $10^{-7}$ were inoculated onto BHI agar (OXOID) and incubated at 36°C for 48 h in aerobic conditions. The results were expressed as CFU/biofilm.

Percentage reduction was determined using the following equation:

%RGt=(G7−Gt)×100/G7,

where %RGt = Percentage of reduction of tested group; G7 = media of CFU/biofilm of negative control (G7); Gt = media of CFU/biofilm of tested group.

### 2.6 Statistical Analysis

Data were statistically analyzed using GraphPad Prism (version 6.0, La Jolla, San Diego, California, United States) software, with a 95% level of confidence. Comparative analyses between the number of CFU/biofilm after each treatment were performed by the Kruskal Wallis test for multiple comparisons. Statistical significance was considered when p values<0.05 were obtained.

## 3 Results

In this study, 28 cellulose acetate disks (four per group) were used to evaluate the activity of aPDT compared with other treatment approaches against a cariogenic microbial biofilm. After treatment, the biofilms from all seven groups presented the three distinct colonial morphotype characteristics for each microorganism present in the cariogenic biofilm.

**Fig. 2:**
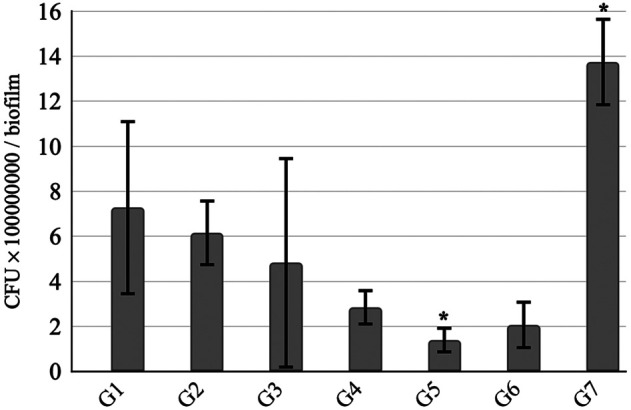
Number of CFU/biofilm after treatments. CFU, colony-forming unity; G1, group 1 (laser 1 Joule); G2, group 2 (laser 4 Joules); G3, group 3 (photosensitizer); G4, group 4 (photosensitizer + laser 1 Joule); G5, group 5 (photosensitizer + laser 4 Joules); G6, group 6 (chlorhexidine); G7, group 7 (distilled water). *Significant statistical difference between groups (p=0.0075).

The results of CFU/biofilm after each treatment are represented in Fig. 2. Following treatments, the number of viable microbial cells per biofilm ranged from $1.40\times 10^{8}$ (G5) to $7.28\times 10^{8}$ (G1), whereas the negative control group (G7) reached $1.38\times 10^{9}$. Compared with G7, all groups (G1 to G6) demonstrated a reduction in microbial load, with the percentage varying from 47.05% (G1) to 89.85% (G5) (Table 1).

**Table 1:** Number of CFU per biofilm and their percent reduction per group compared with negative control (G7).

Group	Treatment	CFU/biofilm	Percent reduction
G1	L 1 J (10 s)	$7.28\times 10^{8}$	47.05^a,b^
G2	L 4 J (40 s)	$6.15\times 10^{8}$	55.27^a,b^
G3	PS	$4.83\times 10^{8}$	64.87^a,b^
G4	PS + L 1 J (10 s)	$2.84\times 10^{8}$	79.35^a,b^
G5	PS + L 4 J (40 s)	$1.40\times 10^{8}$	89.85^a^
G6	Chlorhexidine 0.12%	$2.07\times 10^{8}$	84.93^a,b^
G7	Distilled water	$1.38\times 10^{9}$	—^b^

PS, photosensitizer; L, Laser; J, Joules; CFU, colony-forming units. Different letters in the percentual reduction column represent statistically significant results between groups (p<0.05). G1, group 1 (laser 1 J); G2, group 2 (laser 4 J); G3, group 3 (photosensitizer); G4, group 4 (photosensitizer + laser 1 J); G5, group 5 (photosensitizer + laser 4 J); G6, group 6 (chlorhexidine); G7, group 7 (distilled water).

Groups treated by low-level laser therapy alone, G1 (1 J) and G2 (4 J), achieved CFU/biofilm reductions of 47.05% and 55.27%, respectively. Both showed lower reduction when compared with the photosensitizer-only group (G3), which reached 64.87%. Groups receiving the combination of photosensitizer and low-level laser source, G4 and G5, presented a reduction of 79.35% and 89.85%, respectively. The reduction in G5 (photosensitizer + 4 J) was greater than all groups, including the positive control (G6), thereby rejecting the null hypothesis. Among treatments (G1 to G6), G5 was the only one that showed a significant reduction compared with any group (G7; p=0.0075) (Fig. 2).

## 4 Discussion

In the present study, we used the biofilm model on membrane disks of cellulose acetate filters described by Alviano et al.,^27^ which tested antibiofilm properties of linalool. Other studies also used this model to evaluate antibiofilm properties of coffee extract^28^ and extracts based on Brazilian folk medicine.^29^ We chose this method over biofilm formation in 96-well microtiter plates due to the advantage of washing biofilms without damaging their structure during the treatment. The method we used also ensures that the cells present in the biofilm are recovered when compared with the scraping method used in 96-well microtiter plates. We also observed that microbial cells present in adjacent wells of the microtiter plate are affected by the irradiation (data not shown), which could affect our results because some of the treatments involved irradiation.

Microorganisms in the form of planktonic cells seem to be more susceptible to aPDT when compared with microorganisms organized in biofilms.^30^^–^^32^ Similar to how planktonic and biofilm-associated bacteria behave differently, bacteria in single-species and multispecies systems exhibit distinct behaviors as well.^33^ Although many studies used monospecies biofilms to evaluate antibiofilm strategies,^20^^,^^21^^,^^28^^,^^34^ they appear to be less resistant to aPDT than multispecies biofilms.^35^ In addition, multispecies biofilms allow a better replication of natural oral conditions, considering that human oral biofilms are composed of multispecies microbial communities.^36^ To the best of our knowledge, this is the first laboratory study to compare antibiofilm activity of aPDT, low-level laser irradiation protocols, and the photosensitizer methylene blue alone, against a multispecies biofilm composed of a microbial consortium of two bacteria (*S. mutans* and *L. rhamnosus*) and one yeast (*C. albicans*).

Low-level laser therapy utilizes laser light sources of low energy or intensity, enough to induce stimulation of the target system.^37^ It exhibits a diversity of biostimulatory effects, which include promotion of repair and regeneration, tissue death prevention, and reduction of pain and inflammation.^38^ Red light irradiation enhances mitochondrial ATP production through cytochrome c oxidase, thereby promoting the activation of cell-signaling pathways involved in cellular proliferation and differentiation.^39^

On the other hand, aPDT utilizes light of appropriate wavelength associated with a photosensitizer to form reactive oxygen species, being effective against many pathogenic microorganisms^40^ without promoting resistance, representing a sole or adjunctive therapeutic agent in the treatment of oral infections.^1^

Regarding antibiofilm activity of light irradiation with low-level laser only, we observed a dose-dependent effect, with G2 (4 J) presenting 15.52% less viable microbial cells than G1 (1 J). This dose-dependent effect was observed both in planktonic cells^34^ and biofilms.^17^ Hasanah et al.^34^ observed a reduction in *C. albicans* cells using an 805 nm diode laser at 5 and $10\;J/{\mathrm{cm}}^{2}$. By contrast, Basso et al.,^17^ using an infrared diode laser, indium gallium arsenide phosphide (InGaAsP) (780 of wavelength, 0.4 W of output energy), with different doses (5, 10, and $20\;J/{\mathrm{cm}}^{2}$), demonstrated a dose-dependent effect in the reduction of viable cells present in a *S. mutans* biofilm.

Although our results showed microbial reduction in biofilms using red diode laser (660 nm), InGaAlP, in both tested doses (in a dose-dependent manner), a study^18^ with planktonic *Staphylococcus aureus*, *Escherichia coli*, and *Pseudomonas aeruginosa* cells irradiated with an argon–ion pumped tunable dye laser (630 and 660 nm) and diode laser (810 and 905 nm) showed that aspects such as bacterial species, wavelength, irradiance, and radiant exposure affect bacterial growth, promoting an increase or reduction.

A diversity of protocols has been developed using different combinations of photosensitizers and light sources.^40^ Currently, many researchers have tested a wide range of photosensitizers, such as ones conjugated with inorganic particles^41^; natural extracts and compounds, their synthetic derivatives, and nanoprocessing^40^; and synthetic dyes.^42^ Phenothiazinium dyes are widely used; of these, methylene blue, toluidine blue, rose bengal, dimethyl methylene blue, and new methylene blue are often tested, whereas natural sensitizers include curcumin, hypericin, and flavin derivatives.^42^

In this study, we selected methylene blue as a photosensitizer for aPDT and also evaluated its use alone (without irradiation). Methylene blue properties, such as its positively charged molecule and low molecular mass, promote good interaction with Gram-positive and Gram-negative microorganisms; however, Gram-positive bacteria inactivation is more efficient. These characteristics make this sensitizer suitable for the inactivation of cariogenic microorganisms.^43^

Although extensively used, aPDT with methylene blue allows the use of a wide range of parameters, but the best parameters are not yet defined; so, the energy, exposure time, sensitizer concentration, and severity of infection may have an impact on treatment response.^44^

Compared with the negative control (G7), 0.01% methylene blue solution alone (G3) was responsible for a reduction of 64.87%. The antimicrobial activity of methylene blue and its analogs (methylene green, neutral red, new methylene blue, azure B, dimethyl methylene blue, methylene violet, cresyl violet, acriflavine, and nile blue) were determined against planktonic cells of *S. aureus*, *Staphylococcus epidermidis*, *E. coli*, *Klebsiella pneumoniae*, *Salmonella enterica*, and *C. albicans*. The tested photosensitizers presented antimicrobial activity against *S. aureus*, *S. epidermidis*, and *K. pneumoniae*.^19^ Other studies using methylene blue and other photosensitizers also associated antimicrobial and antibiofilm properties with these substances.^20^^–^^23^

The reduction observed for methylene blue was higher than the one achieved through 1 J and 4 J laser irradiation alone. By contrast, a study comparing methylene blue in the same concentration of 0.01%, LED irradiation (660 nm, $330\;\mathrm{mW}/{\mathrm{cm}}^{2}$), and the combination of both showed a more significant reduction in metabolic activity in a multispecies subgingival biofilm in the LED alone than in the non-LED application group.^22^ This divergence may be attributed to factors such as differences in biofilm composition and in light parameters.

A study with planktonic *C. albicans* showed statistical differences between laser irradiation (660 nm, 40 mW, $10\;J/{\mathrm{cm}}^{2}$) and methylene blue alone, with greater reduction in the irradiated cells group.^20^ This contrast with our findings could be justified by the difference in the parameters of irradiation dose applied. Another study demonstrated no difference between a diode laser (660-nm, 100 mW) and methylene blue against *S. mutans*.^21^ Soares et al.^23^ submitted a double species biofilm (*S. mutans* and *C. albicans*) to 5 (the same used in our study), 15, and 30 min of pre-irradiated time with methylene blue. They observed that the increase in pre-irradiation time was important in the reduction of biofilm microbial cells.^23^

Despite divergences regarding the antimicrobial properties of light, reducing or increasing^17^^,^^18^ microbial count, it seems that the combined action of light and photosensitizer (aPDT) achieves better results even when compared with chlorhexidine or antifungal substances such as nystatin.^20^ This aligns with our findings, in which G5 (aPDT: PS + L 4 J) was responsible for the greatest microbial reduction observed in this study, even when compared with G6 (chlorhexidine). In addition, G5 was the only group statistically different from any other group (G7: distilled water).

The dose-dependent nature of aPDT’s antimicrobial effects in biofilms has been previously demonstrated.^45^ In our study, the aPDT group with a higher dose (G5) presented a higher antibiofilm effect than the aPDT group with a lower dose (G4), even though it was not statistically significant.

Moreover, aPDT presents the benefit of not only eliminating bacteria in biofilms but also leading to biofilm disruption;^35^ in addition, it does not contribute to the development of microbial resistance.^35^ Chlorhexidine mouthwash, used as a positive control in this study, is often used to reduce *S. mutans* and/or oral microbial load due to its proven bactericidal effects; however, its continuous use may cause complications such as dysgeusia, discoloration of teeth, and restorations.^21^

Despite the ease of mechanical removal of this type of biofilm through dental brushing, aPDT can serve as a valuable adjunct therapy for microbial load depletion in various dental procedures, such as prophylactic bacterial reduction prior to surgery and biofilm control in patients with mental or behavioral disorders or who reject traditional methods. Another possible application could be plaque control in patients presenting oral mucositis, herpetic lesions, immune-mediated diseases, and other painful conditions that may hinder dental/oral hygiene, especially in hospital care.

This study has a few limitations due to its *in vitro* nature, considering that the efficacy exhibited by aPDT *in vitro* may not be fully translated into *in vivo* trials.^46^ In addition, this study is focused on a biofilm involved in initial caries development, not including microorganisms found in later stages of this disease. Thus, we acknowledge that further assays could be performed in the near future, such as different irradiation times; biocompatibility; cellular uptake of methylene blue and its penetration through biofilm; efficacy of double/successive aPDT; microbial recovery and development of resistance after treatment; and impact on the expression of virulence factors.

## 5 Conclusion

aPDT represents an important therapy to be used as an antibiofilm adjunct treatment, leading to a significant reduction of microbial cells within a cariogenic biofilm model.

## Data Availability

Data sharing is not applicable to this article as no new data were created or analyzed.
